# Exposure to Maternal Pre‐ and Postnatal Psychological Distress: Associations With Brain Structure in 5‐Year‐Old Children

**DOI:** 10.1002/hbm.70683

**Published:** 2026-11-06

**Authors:** Elmo P. Pulli, Hilyatushalihah K. Audah, Amira Svensk, Eeva Eskola, Elisabeth Nordenswan, Aylin Rosberg, Silja Alvajärvi, Pauliina Juntunen, Isabella L. C. Mariani Wigley, Venla Kumpulainen, Eero Silver, Anni Copeland, Ekaterina Saukko, Harri Merisaari, Riikka Korja, Eeva‐Leena Kataja, Linnea Karlsson

**Affiliations:** ^1^ FinnBrain Birth Cohort Study, Turku Brain and Mind Center, Department of Clinical Medicine University of Turku and Turku University Hospital Turku Finland; ^2^ Centre for Population Health Research Turku University Hospital and University of Turku Turku Finland; ^3^ Department of Psychiatry University of Turku and Turku University Hospital Turku Finland; ^4^ The Centre of Excellence for Learning Dynamics and Intervention Research (InterLearn) University of Turku Turku Finland; ^5^ Department of Psychology and Speech‐Language Pathology University of Turku Turku Finland; ^6^ Department of Psychology Åbo Akademi University Turku Finland; ^7^ Department of Pediatric Neurology Turku University Hospital Turku Finland; ^8^ Department of Radiology Turku University Hospital and University of Turku Turku Finland; ^9^ Department of Child Psychiatry Turku University Hospital and University of Turku Turku Finland; ^10^ Department of Public Health University of Turku and Turku University Hospital Turku Finland

**Keywords:** magnetic resonance imaging (MRI), maternal stress, postnatal stress, prenatal stress, structural brain development, voxel‐based morphometry (VBM)

## Abstract

Maternal mental health is an important contributor to child neurodevelopment. While there are multiple studies on prenatal exposure, early postnatal exposure has received little attention in neuroimaging research. Five‐year‐old children (*n* = 161) were recruited from the FinnBrain Birth Cohort study. Maternal distress was assessed using questionnaires on depressive and anxiety symptoms at 14, 24, and 34 gestational weeks and postnatally at 3, 6, and 24 months. T1‐weighted structural images were processed using a voxel‐based morphometry pipeline to map associations between maternal distress exposure and regional gray matter (GM) volumes. Whole‐brain voxel‐wise general linear models were used, accounting for potential confounders. Additionally, a latent class mixed model approach was used to model longitudinal trajectories of distress exposure. We found widespread associations between maternal postpartum distress symptoms and offspring brain morphology. Postnatal distress at 3 months was negatively associated with GM volumes in the left supplementary motor cortex, right cerebellum/fusiform gyrus, and left anterior insula/frontal operculum. Postnatal distress at 6 months was positively associated with GM volumes in the right cuneus and lingual gyrus, right insula/operculum, and right inferior occipital gyrus, while distress at 24 months was negatively associated with GM volumes in the left inferior/middle frontal gyrus. There were no structural brain differences between the latent classes. This study provides support for hypotheses proposing that early‐life exposure to maternal distress can influence the structural development of the brain. It highlights the role of the early postnatal period and calls for further research into this so far overlooked period and the developmental pathways that explain these associations.

## Key Points

- We examined the associations between exposure to maternal distress at multiple pre‐ and postnatal time points and brain structure at 5 years of age.
- Statistically significant associations were only observed between postnatal distress exposure and brain structure.
- This study highlights the role of the early postnatal period and calls for further research into this so far overlooked period and the developmental pathways that explain these associations.

## Graphical abstract

This study provides support for hypotheses proposing that early‐life exposure to maternal distress can influence the structural development of the brain. Furthermore, it highlights the role of the early postnatal period and calls for further research into this so far overlooked period and pathways that explain the associations.

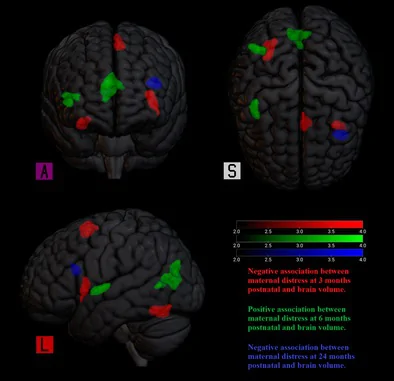

## 1 Introduction

Maternal pre‐ and postnatal mental distress (e.g., depressive or anxiety symptoms) may have significant, enduring, and potentially irreversible effects on offspring development (Lautarescu, Craig, and Glover 2020; Meaney 2018; Van den Bergh et al. 2020). Both pre‐ and postnatal distress share similarities in their potential long‐term outcomes (Rogers et al. 2020) which could reflect increased genetic vulnerability (Meng et al. 2024; Wray et al. 2018). It is still useful to study the timing of exposure, as pre‐ and postnatal distress exposure involve different mechanisms. Prenatal distress can affect fetal development via biological pathways such as perturbation of the hypothalamic–pituitary–adrenal axis function, increased inflammation (Graham et al. 2022), and epigenetic modifications of the placenta (Bind 2022), while postnatal maternal distress can affect the offspring through psychosocial and environmental factors, such as maternal sensitivity (Lefkovics et al. 2014) and attachment patterns (Barnes and Theule 2019). The environment can affect the brain through neural mechanisms such as activation‐dependent plasticity, mediated by brain‐derived neurotrophic factor (Fahnestock et al. 2012; Hsiao et al. 2014; Miranda et al. 2019). As some of the mediating mechanisms are different, pre‐ and postnatal exposures could have different effects on offspring structural brain development, which can be studied using magnetic resonance imaging (MRI).

Prior neuroimaging research has focused more on maternal prenatal than postnatal distress. Common measures include maternal depressive, anxiety, and stress symptoms based on questionnaires (Lautarescu, Craig, and Glover 2020). Furthermore, structural neuroimaging studies have typically focused on subcortical structures (Bezanson et al. 2023; Donnici et al. 2023; Favaro et al. 2015; Qiu et al. 2013), especially the amygdala (Acosta et al. 2019, 2020; Donnici et al. 2021; Favaro et al. 2015; Lee et al. 2019; Lehtola et al. 2020; Lupien et al. 2011; Moog et al. 2021; Wen et al. 2017; Wu et al. 2021) and the hippocampus (Favaro et al. 2015; Moog et al. 2021; Wang et al. 2018). Previous amygdala studies have found positive (Groenewold et al. 2022; Wen et al. 2017) and negative (Acosta et al. 2020; Favaro et al. 2015; Wu et al. 2021) associations between prenatal maternal distress and amygdala volume as well as different associations based on child sex (Acosta et al. 2019; Lehtola et al. 2020; Wen et al. 2017), nonlinear relationships (Donnici et al. 2023), or no associations at all (Moog et al. 2021; Triplett et al. 2022). Hippocampal findings have been similarly inconsistent. Some studies reported smaller left (Moog et al. 2021), smaller right (Weiner et al. 2024), and larger bilateral (Groenewold et al. 2022) hippocampal volume in newborns associated with maternal prenatal stress. Other studies have focused on gene–environment interactions influencing right hippocampal volume (Qiu et al. 2017; Wang et al. 2018), while other studies found no significant hippocampal volume differences (Favaro et al. 2015; Lehtola et al. 2020; Triplett et al. 2022). Prenatal distress has also been associated with white matter structure (Lautarescu, Pecheva, et al. 2020) and brain function in the offspring (Wu et al. 2024).

Existing research indicates that prenatal exposure to maternal psychological distress is associated with alterations in offspring cortical brain development (Buss et al. 2010; Davis et al. 2020; El Marroun et al. 2016; Sandman et al. 2015; Wei et al. 2022). These associations have been observed across multiple cortical regions, with frontal (Buss et al. 2010; Davis et al. 2020; El Marroun et al. 2016; Sandman et al. 2015; Wei et al. 2022) and temporal (Buss et al. 2010; Davis et al. 2020; Wei et al. 2022) regions emerging most consistently, though some report differences in parietal (Buss et al. 2010; Wei et al. 2022) and occipital (Buss et al. 2010; El Marroun et al. 2016) regions. In some cases, the direction of the associations differed between sexes (Wei et al. 2022). Although cortical development has received comparatively less attention than subcortical development, the available findings broadly suggest that prenatal maternal distress is associated with structural development across widespread cortical regions, particularly those involved in higher‐order cognitive processes.

Postnatal maternal mental health has received less attention in neuroimaging studies, although it has adverse effects on child development comparable to prenatal exposure (Rogers et al. 2020). Two studies in 2‐ to 3‐year‐olds found no associations between postnatal depression exposure and CT or area (El Marroun et al. 2016; Pellowski et al. 2023), while one of them did find smaller amygdala volumes in those exposed to postnatal depression (Pellowski et al. 2023). In older children, exposure to maternal depression has been associated with: smaller right superior frontal CT in 2.6‐ to 5.1‐year‐olds (Lebel et al. 2016), smaller fusiform gyrus volume in 7‐ to 15‐year‐olds (Koc et al. 2023), and total GM volume in 10‐year‐olds (Zou et al. 2019). Overall, the cortical structural neuroimaging literature on the associations of early‐life postnatal maternal distress exposure is limited and the results are limited to individual ROIs that do not replicate between studies.

The aim of this study was to investigate the associations between pre‐ and postnatal maternal stress and brain structure in 5‐year‐old children. Distress was measured longitudinally utilizing questionnaire data on depressive and anxiety symptoms from gestational weeks (GWs) 14, 24, and 34 as well as from 3, 6, and 24 months of child age postnatally. We expected to find: (1) significant differences in GM volumes in fronto–temporal regions; and (2) alterations in amygdalar and hippocampal volumes in those exposed to pre‐ and/or postnatal distress. The 5‐year measurement point has been rarely covered in earlier studies, and it is one of the earlier opportunities to examine the neural correlates of early postnatal exposure to maternal distress, as toddlerhood is a particularly challenging time to collect MRI data. As previous studies in this age group and studies combining pre‐ and postnatal distress symptoms are scarce, we reported exploratory whole‐brain VBM analyses.

## 2 Materials and Methods

This study adheres to the ethical guidelines in the 1975 Declaration of Helsinki and has been approved by the joint Ethics Committee of the University of Turku and the Hospital District of Southwest Finland (ETMK:31/180/2011). This study follows the Strengthening the Reporting of Observational Studies in Epidemiology (STROBE) guidelines (Vandenbroucke et al. 2014), checklist in Table S1.

### 2.1 Participants

The participants of the present study are mother–child dyads (*n* = 161) drawn from the FinnBrain Birth Cohort study (www.finnbrain.fi) (Karlsson et al. 2018). Children who underwent neuroimaging during their 5‐year follow‐up visit (*n* = 203, 55.7% boys, mean age 5.39 [SD 0.13], range 5.08–5.79 years) were eligible to participate. Exclusion criteria for the MRI included the following: premature birth (< 35 GW, or < 32 GW for those with exposure to maternal prenatal synthetic glucocorticoid treatment); developmental sensory anomaly or disability (e.g., blindness); long‐term medical conditions (e.g., epilepsy, autism spectrum disorders); current ongoing medical examinations or clinical follow‐ups; usage of daily medications; history of head trauma; routine MRI contraindications. For the purposes of this study, all participants born < 37 GW were excluded. More details on the cohort and recruitment process in Supporting Information. The final sample consisted of 161 participants with high‐quality structural imaging data. A flowchart of the participant selection process is presented in Figure 1. Participant characteristics are presented in Table 1.

**FIGURE 1:**
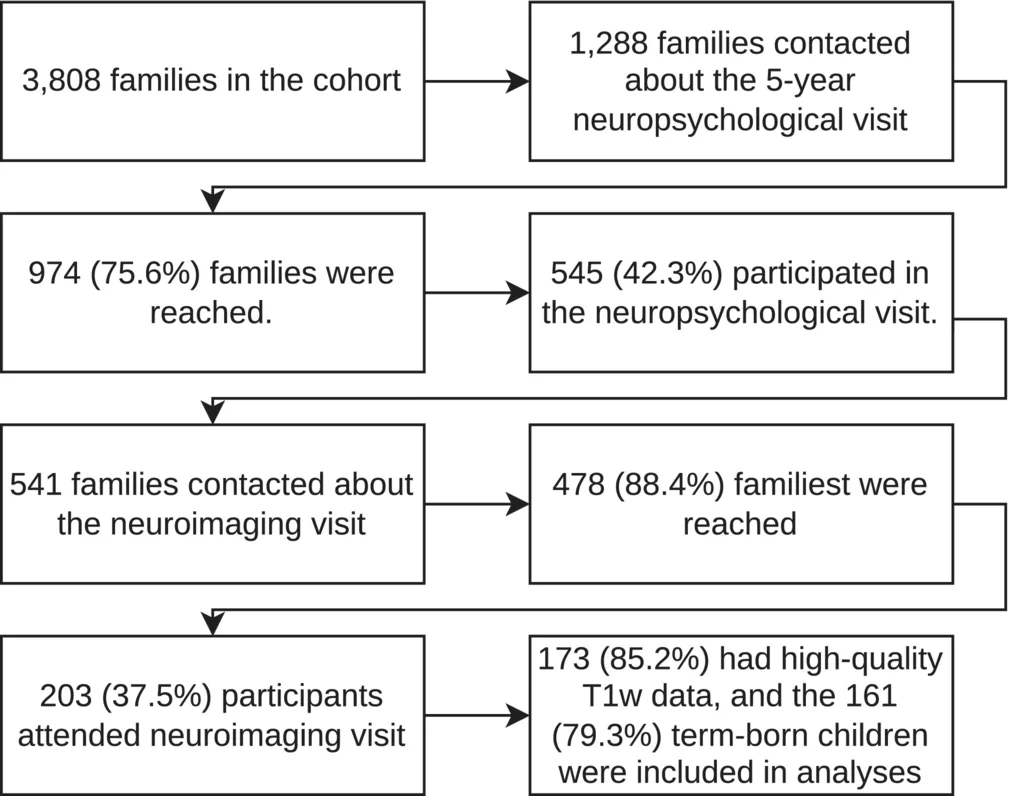
A flowchart of the recruitment process of participants. T1w = T1‐weighted structural imaging.

**TABLE 1:** Demographic variables and distress scores of participating mothers and children.

	Mean	SD	Min	Max
Maternal age at term (years)	30.95	4.68	19.10	41.31
Maternal body mass index before pregnancy	24.24	4.21	17.48	41.95
Child gestational age at birth (weeks)	40.01	1.23	37.14	42.29
Child age at scan (years)	5.41	0.13	5.09	5.79
Ponderal index	14.06	1.17	11.21	17.63
14 gestational weeks composite distress score	7.35	7.20	0	39
24 gestational weeks composite distress score	7.87	7.21	0	34
34 gestational weeks composite distress score	7.59	7.60	0	39
3 months composite distress score	6.56	6.80	0	30
6 months composite distress score	7.74	8.15	0	43
24 months composite distress score	7.23	8.09	0	42.67
14 gestational weeks depressive symptoms score	4.38	3.96	0	22
24 gestational weeks depressive symptoms score	4.50	4.01	0	21
34 gestational weeks depressive symptoms score	4.61	4.40	0	20
3 months depressive symptoms score	4.19	3.86	0	19
6 months depressive symptoms score	4.77	4.46	0	22
24 months depressive symptoms score	4.56	4.61	0	21
14 gestational weeks anxiety symptoms score	2.97	3.66	0	17
24 gestational weeks anxiety symptoms score	3.40	3.80	0	19
34 gestational weeks anxiety symptoms score	2.98	4.02	0	19
3 months anxiety symptoms score	2.38	3.51	0	17
6 months anxiety symptoms score	2.97	4.34	0	24
24 months anxiety symptoms score	2.67	4.19	0	26.67

	*N*	%
Mother		
Education level		
Low	34	24.4
Middle	42	26.9
High	76	48.7
Maternal monthly income (in €)		
< 1500	50	31.1
1501–2500	88	54.7
2501–3500	14	8.7
> 3501	3	1.9
Missing	6	3.7
Alcohol use during pregnancy		
Yes, before learning about pregnancy	21	13.0
Yes, continued after learning about pregnancy	24	14.9
No	115	71.4
Not reported/missing	1	0.6
Tobacco product use during pregnancy		
Yes	11	6.8
No	150	93.2
Illicit drug use during pregnancy		
No	152	94.4
Not reported/missing	9	5.6
SSRI or SNRI use during first trimester		
Yes	3	1.9
No	148	91.9
Missing	10	6.2
Community of origin		
Finnish	152	94.4
Other or mixed	4	2.5
Missing	5	3.1
Child		
Sex		
Female	72	44.7
Male	89	55.3
Gestational age at birth, weeks		
≥ 37 (Term)	161	100
< 37 (Preterm)	0	0
NICU admission		
Yes	24	14.9
No	136	84.5
Not reported/missing	1	0.6
Obstetric risk		
Present	24	14.9
Absent	136	84.5
Not reported/missing	1	0.6

*Note:* Ponderal index was calculated using the following formula: weight in kilograms divided by height in meters cubed. Height and weight were acquired during the neuroimaging visit. The participants kept indoor clothes on during the weighing. Maternal education data were combined from questionnaire data from gestational Week 14 or 5 years of child age by choosing the highest degree reported. The three classes are: Low = upper secondary school or vocational school or lower, Middle = university of applied sciences, High = university. The data for maternal monthly income estimate, SSRI/SNRI and illicit drug use are from questionnaires at gestational Week 14. Obstetric risk was defined as diagnosis (according to ICD‐10, during pregnancy) for O12 (gestational edema and proteinuria without hypertension), O13 (gestational hypertension without significant proteinuria), O14 (severe pre‐eclampsia), O24 (diabetes mellitus in pregnancy, childbirth, and the puerperium), O46 (antepartum hemorrhage, not elsewhere classified), or O99.0 (anemia complicating pregnancy, childbirth and the puerperium). Sex, birth weight, maternal body mass index before pregnancy, and smoking data (combined with questionnaire data) were retrieved from the wellbeing services county of Southwest Finland (VARHA) records.

Abbreviations: NICU = neonatal intensive care unit, SNRI = serotonin and norepinephrine reuptake inhibitor, SSRI = selective serotonin reuptake inhibitor.

### 2.2 Procedures

Following recruitment, participating mothers provided demographic information and completed a set of standardized and internationally validated self‐report questionnaires regarding depressive and anxiety symptoms three times during pregnancy (14, 24, and 34 GW) and three times in the postnatal period (3, 6, and 24 months). MRI and neuropsychological data were collected at age 5 years.

### 2.3 Measures

Maternal depressive symptoms were assessed using the Finnish version of the Edinburgh Postnatal Depression Scale (EPDS) (Cox et al. 1987). The clinical cut‐off threshold for potential depression is ≥ 10 points. Maternal anxiety symptoms were evaluated using the Finnish version of the Symptom Checklist‐90‐Revised (SCL) (Derogatis 1994). For the purposes of this study, only the anxiety subscale was utilized. The 10‐item scale assesses signs of nervousness, tension, and feelings of panic. The questionnaires are described in more detail in Supporting Information.

Scores from both the EPDS and SCL were added together to create a unified measure of maternal psychological distress, the composite distress score (CDS). The rationale behind creating a combined, overall measure of distress is twofold: (1) study population was drawn from the general population and consequently, individual scores were relatively low, and (2) symptoms of anxiety and depression are often comorbid (Kessler et al. 2015; Lamers et al. 2011). A correlation matrix of EPDS and SCL scores is presented in Table S2. Thus, combining the measurements may provide a more robust tool to measure overall distress. Previous authors have used composite scores (Lehtola et al. 2020; Rajasilta et al. 2023) or principal component approaches (Lahti‐Pulkkinen et al. 2024) in an attempt to grasp the overlap of various distress measures. The EPDS and SCL scores were not standardized while calculating the CDS, as that would make the effect sizes not comparable with other studies. Pearson correlations between non‐standardized and standardized CDS = 1.000 at each time point; Spearman correlation ≥ 0.998 at each time point. Main Model 1 (see Section 2.7) was run with both standardized and non‐standardized values, giving the same significant results (same peak coordinates, 5% difference in cluster size). All following analyses were run with non‐standardized values, only.

There were missing maternal questionnaire data at various follow‐up points (14 GW *n* = 7, 24 GW *n* = 6, 34 GW *n* = 6; 3 months *n* = 16, 6 months *n* = 30, 24 months *n* = 42), mean imputation of overall sum scores was used to replace the missing data in such cases (mean of the final MRI sample). With missing covariate data, mean imputation was used for maternal prepregnancy body mass index (one missing case) and mode imputation with categorical variables (missing data indicated in Table 1). Missing data are visualized in Figure S1.

### 2.4 Cognitive Performance Measurement

The neuropsychological visits are described in more detail in Supporting Information. Neurocognitive testing at 5 years of age included assessments of the child's general cognitive ability using the Finnish version of WPPSI‐III subtests Block Design, Matrix Reasoning, and Similarities. WPPSI‐III is a standardized and widely used measure of cognitive ability in young children from ages 2 years and 6 months to 7 years and 3 months (Wechsler 2009). In this study, a composite sum score of nonverbal ability (performance intelligence quotient = PIQ) was estimated using two subtests: the Block Design task measuring visuospatial ability and the Matrix Reasoning task measuring visual abstract reasoning. The standardized scale scores corresponding to the raw scores of the subtests were based on Finnish norms and result in a mean of 10, reflecting standardized mean performance in the population at each age. Additionally, analyses of the subtests were conducted separately to get further information on the possible subtest driving the findings.

### 2.5 Image Acquisition

The neuroimaging visit is described in our earlier publications (Pulli et al. 2022) and in the Supporting Information. Participants were scanned on a 3T MRI system (MAGNETOM Skyra fit; Siemens Medical Solutions, Erlangen, Germany) equipped with a 20‐channel head/neck coil. To speed up the acquisition process, the Generalized Auto‐calibrating Partially Parallel Acquisition technique was employed, with a parallel acquisition technique factor of two applied to all image acquisitions. The MRI data collection was completed within a scan protocol lasting < 60 min. For the purposes of the current study, high‐resolution 3D T1‐weighted images using the magnetization prepared rapid gradient echo technique were acquired. This acquisition used the following sequence parameters: repetition time = 1900 ms, echo time = 3.26 ms, inversion time = 900 ms, flip angle = 9 degrees, voxel size = 1.0 × 1.0 × 1.0 mm^3^, and field of view 256 × 256 mm^2^.

### 2.6 Data Preprocessing and Statistical Analyses

#### 2.6.1 Voxel‐Based Morphometry

VBM is a method that enables researchers to study regional GM volume differences between various groups of participants on a voxel‐by‐voxel basis. In our study, the Statistical Parametric Mapping (SPM, version 25.01.02; RRID:SCR_007037) (Tierney et al. 2025) software running on MATLAB R2024b (MathWorks, MA) was used to identify alterations in regional GM volumes associated with maternal pre‐ and postnatal distress.

Preprocessing of the data was done with the Computational Anatomy Toolbox (CAT12, version r1363; RRID:SCR_019184) (Gaser et al. 2024). A standard preprocessing pipeline, encompassing normalization, segmentation, modulation, quality assessment, and smoothing, was applied with the following specifications: the default tissue probability map provided in SPM was used, affine regularization was done with the European brains template, inhomogeneity correction was medium, the affine preprocessing option was “rough,” local adaptive segmentation was performed at medium, skull‐stripping was done via the Graph cuts (GCUT) approach, voxel size for normalized images was set to 1.5 mm, and internal resampling for preprocessing was fixed at 1.0 mm. Dartel was used for spatial registration, and the MNI152 template was used as Dartel template. For statistical analyses, images were smoothed with 8 mm full width at half maximum (FWHM) gaussian smoothing kernel.

Adequate quality of MNI space registration was assessed visually (by author E.P.P.) using the warped, normalized outputs (“wm” images). A satisfactory alignment of global landmarks and subcortical structures as well as lack of excessive local errors were confirmed for all 161 participants.

### 2.7 Statistics

Data preparation, missing data imputation, assumption checks, and post hoc mediation analyses were performed using IBM SPSS (Version 31.0; IBM Corporation). Longitudinal trajectory modeling was performed using R version 4.6.0 with RStudio (Version 2026.05.0+218) (RStudio Team 2020). All analyses were performed two‐tailed.

We performed whole‐brain general linear models in SPM12 with the CDS at 14, 24, and 34 GW and at 3, 6, and 24 months postpartum as covariates of interest. Based on findings of previous FinnBrain publications on the same age group (Silver et al. 2022), the following key covariates were included in all analyses: child age at scan, sex, and ponderal index, maternal age at term and education level (two classes: (1) any university degree and (2) other degrees). Additional covariates included CDS points from other ages and early developmental exposures that could potentially affect individual development. For all CDS measurements, three VBM analyses were performed (independent variable of interest is the CDS from each time point):

– Model 1—controlling for key covariates: Brain structure ~ CDS + Age at scan + Sex + Ponderal index + Maternal age at term + Maternal education.
– Model 2—controlling for other CDS time points (example model from 3 months postnatal): Brain structure ~ CDS at 3 months postnatal + Age at scan + Sex + Ponderal index + Maternal age at term + Maternal education + Combined CDS of the other two postnatal measurements + Combined CDS of all prenatal measurements.
– Model 3—controlling for all covariates (example model from 3 months postnatal): Prenatal ROI eigenvalues ~ CDS at 3 months postnatal + Age at scan + Sex + Ponderal index + Maternal age at term + Maternal education + Prenatal alcohol exposure + Prenatal smoking exposure + Prenatal selective serotonin reuptake inhibitor or serotonin and norepinephrine reuptake inhibitor exposure + Obstetric risk + Maternal prepregnancy body mass index + Neonatal intensive care unit admission + Combined CDS of the other two postnatal measurements + Combined CDS of all prenatal measurements.

See Table 1 for missing data and more details on covariates. For each main analysis result, we report whether the results remained in the sensitivity analyses. All analyses were run at a voxel threshold of 0.001 uncorrected (Silver et al. 2011), and clusters were considered statistically significant at FDR‐corrected *p* value < 0.05 (calculated automatically by SPM). Furthermore, we report whether the results survive a further Bonferroni correction over 36 comparisons (3 models for 6 time points, with 2 contrasts each). Bonferroni corrected *p* value = 0.05/36 = 0.0014.

As a sensitivity analysis, we repeated the analyses for all contrasts with statistically significant results with the following four changes (using the same covariates):

1. Complete data analysis: The participants with missing data at the time point in question were excluded. The resulting sample sizes were 145 for 3 months, 131 for 6 months, and 119 for 24 months postpartum.
2. Winsorized analysis: The analyses were run with winsorized data (5th and 95th percentiles) to limit the effect of outliers. In all cases, the lowest value remained at zero. Therefore, in effect, winsorization limited the effect of high‐distress outliers on the results.
3. EPDS: CDS was replaced by EPDS score from the same age to see whether the results were driven by depressive symptoms.
4. SCL: CDS was replaced by SCL score from the same age to see whether the results were driven by anxiety symptoms.

#### 2.7.1 Longitudinal Trajectories

Longitudinal distress trajectories were examined using latent class mixed models (LCMMs) implemented with the hlme() function from the R package “lcmm” (version 4.6.0). The original distress assessment time points were rescaled to continuous time intervals in weeks from the baseline measurement at gestational Week 14, rounded to the closest integer (0, 10, 20, 39, 52, and 129 weeks). The whole sample (*n* = 161) was used in the model. Distress scores were not imputed. To determine the optimal number of latent classes, a series of models ranging from a single‐class solution to a five‐class solution was systematically evaluated. A linear growth shape was specified. To capture both population‐level trends and individual variability, models included both fixed and random intercepts and slopes, allowing individual trajectories to deviate from their assigned class average. For multi‐class models, class‐specific fixed intercepts and slopes were estimated to capture the unique trajectory shapes defining each latent subpopulation. To avoid local maxima and ensure the global maximum likelihood solution was achieved, a grid search strategy was utilized (gridsearch function). Multi‐class models were run with 200 random departures and a maximum of 50 iterations, using the parameters from the stable 1‐class model as initial values. A random seed of 1234 was specified to ensure exact reproducibility. We selected the 2‐class model based on model class sizes, fit indices, entropy, and interpretability of the resulting trajectories (model performance information in Table S3). Individuals were assigned to their most likely latent class using the highest posterior probability assignment rule. Model‐implied trajectories were generated and plotted alongside empirical individual data to visually validate how well the selected model captured the underlying data patterns. The statistical analysis was performed in SPM with the two‐sample *t*‐test option; equal variances were not assumed. Age and sex were included as covariates.

#### 2.7.2 Post Hoc Analyses

The MarsBaR toolbox (RRID:SCR_009605) (Brett et al. 2002) within SPM12 was used to extract significant clusters of GM and their corresponding eigenvalues for mediation analyses. The significant clusters from main analyses were included as mediators between distress exposure and PIQ. The mediation analyses were performed with the Hayes' PROCESS macro (Hayes 2022), using 5000 bootstraps (seed 1234) and the same covariates as in the main models (with the exception of dropping SSRI/SNRI exposure from the “all covariates” models, as it caused issues with bootstrapping). Assumptions of approximately linear relationships between the moderator, dependent, and independent variables, homoscedasticity, and normal distribution of residuals were confirmed visually. There was no problematic collinearity between the covariates (VIF < 2.2 for all variables).

## 3 Results

### 3.1 Voxel‐Based Morphometry Results

#### 3.1.1 Controlling for Key Covariates

Statistically significant results were only seen at 3 months postpartum. GM volume in the left supplementary motor cortex was negatively associated with CDS at 3 months postpartum (*p* = 0.028, cluster size 337, peak −8, 8, 58), which was not significant after Bonferroni correction. The result is visualized in Figure 2. The cluster did not remain significant using only complete data (*p* = 0.075, cluster size 256, peak −8, 8, 58). The cluster did remain significant with winsorized data analyses (*p* = 0.001, cluster size 744, peak −8, 8, 58). Winsorized analyses also revealed two new clusters (Figure S2). EPDS analyses revealed the same significant cluster (*p* = 0.009, cluster size 422, peak −8, 8, 58), while SCL analyses revealed a different cluster that was negatively associated with CDS at 3 months postpartum (Figure S3).

**FIGURE 2:**
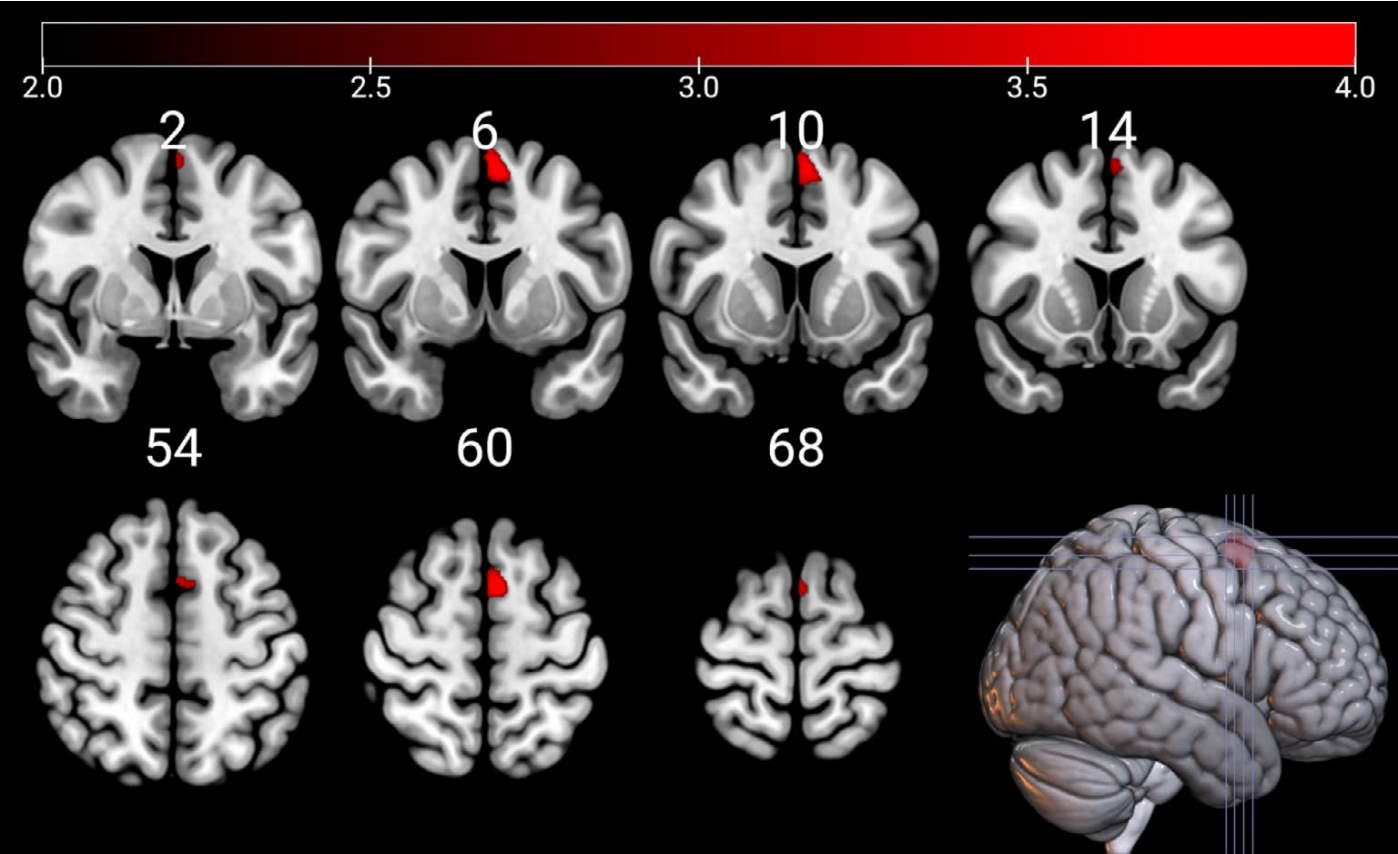
Negative association between maternal distress symptoms at 3 months postpartum and regional volume in the left supplementary motor cortex (controlling for key covariates). Only statistically significant regions at *p* < 0.001, false discovery rate corrected at cluster level (*p* < 0.05) are displayed. Color indicates *t*‐value. Images are in radiological orientation. Coordinates (indicated with white numbers) are in MNI space. The figure was created with MRIcroGL.

#### 3.1.2 Controlling for Other Distress Time Points

Statistically significant results were found at all postpartum time points.

CDS at 3 months postpartum was negatively associated with GM volume in the following regions: the left supplementary motor cortex (*p* = 0.027, cluster size 290, peak −4, 8, 58), the right cerebellum/occipital fusiform gyrus (*p* = 0.010, cluster size 467, peak 32, −66, −20), and the left anterior insula/frontal operculum (*p* = 0.018, cluster size 353, peak −39, 10, 2). None of the clusters remained significant after a Bonferroni correction. Clusters are visualized in Figure 3. None of these clusters remain significant in complete data analyses. All of the clusters remained significant in winsorized analyses: the left supplementary motor cortex (*p* = 0.001, cluster size 778, peak −4, 8, 58), the right cerebellum/occipital fusiform gyrus (*p* = 0.001, cluster size 662, peak 32, −66, −20), and the left anterior insula/frontal operculum (*p* = 0.001, cluster size 715, peak −39, 10, 4). The winsorized analyses also revealed an additional cluster in the left medial orbital gyrus (*p* = 0.001, cluster size 594, peak −20, 12, −22). EPDS analyses showed a significant cluster in left supplementary motor area (*p* = 0.020, cluster size 358, peak −6, 6, 58), while the other two were no longer significant. SCL analyses showed a significant cluster in the right cerebellum/occipital fusiform gyrus (*p* = 0.004, cluster size 474, peak 26, −72, −14). There were also two new clusters in the SCL analyses, visualized in Figure S4.

**FIGURE 3:**
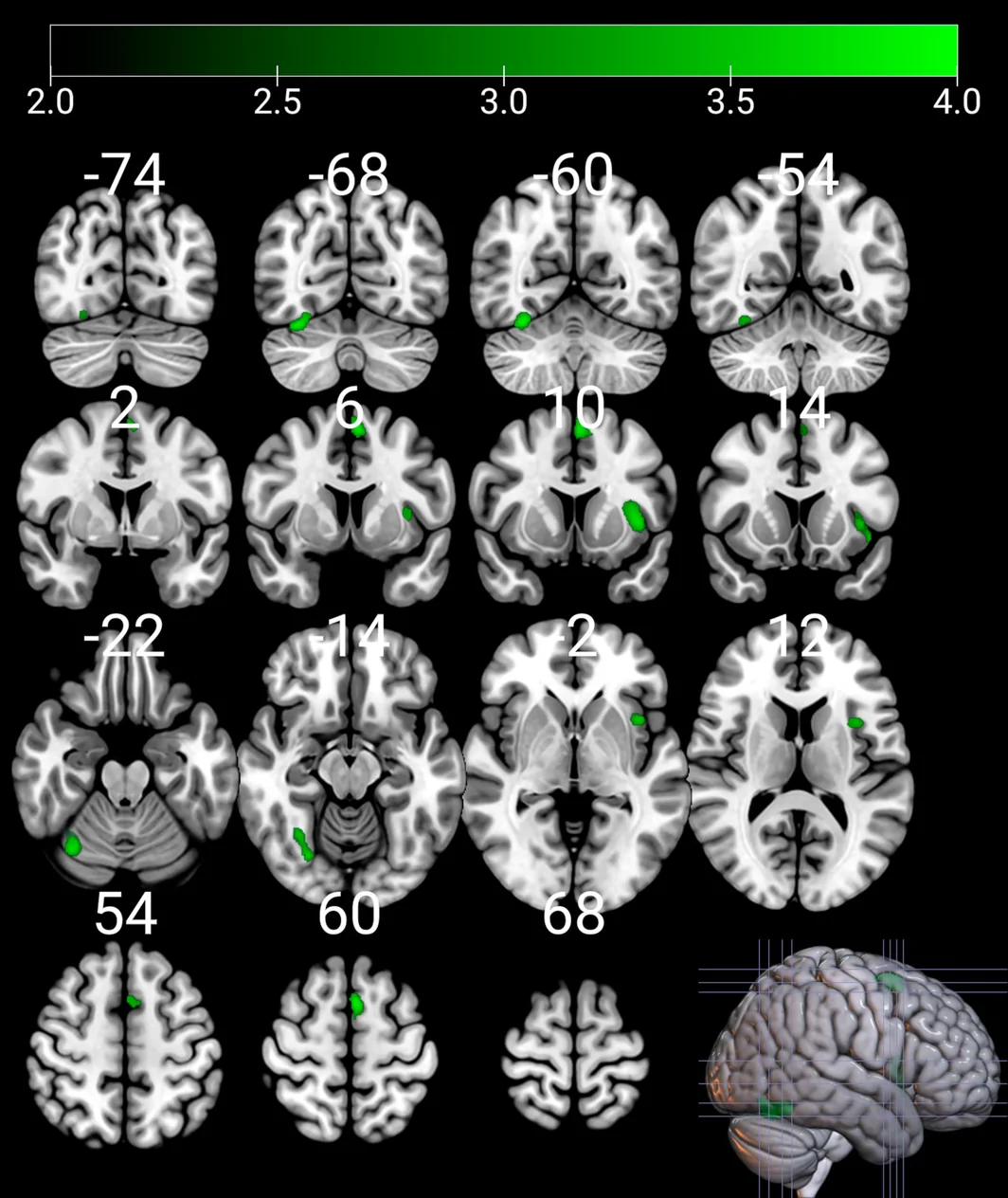
Negative associations between maternal distress symptoms at 3 months postpartum and regional volume in the left supplementary motor cortex, the right cerebellum/occipital fusiform gyrus, and the left anterior insula/frontal operculum (controlling for key covariates and maternal distress symptoms at other ages). Only statistically significant regions at *p* < 0.001, false discovery rate corrected at cluster level (*p* < 0.05) are displayed. Color indicates *t*‐value. Images are in radiological orientation. Coordinates (indicated with white numbers) are in MNI space. The figure was created with MRIcroGL.

CDS at 6 months postpartum was positively associated with GM volume in the right medial occipital lobe, including the cuneus and calcarine regions, extending to the left hemisphere (*p* < 0.001, cluster size 878, peak 9, −78, 18). This cluster remained significant after a Bonferroni correction. The cluster is visualized in Figure 4. The result remains significant in the complete data analyses (*p* = 0.001, cluster size 655, peak 6, −76, 26) and winsorized analyses (*p* = 0.002, cluster size 558, peak 8, −78, 20). EPDS analyses revealed the same cluster (*p* < 0.001, cluster size 2019, peak 9–78, 16). SCL analyses revealed no significant results.

**FIGURE 4:**
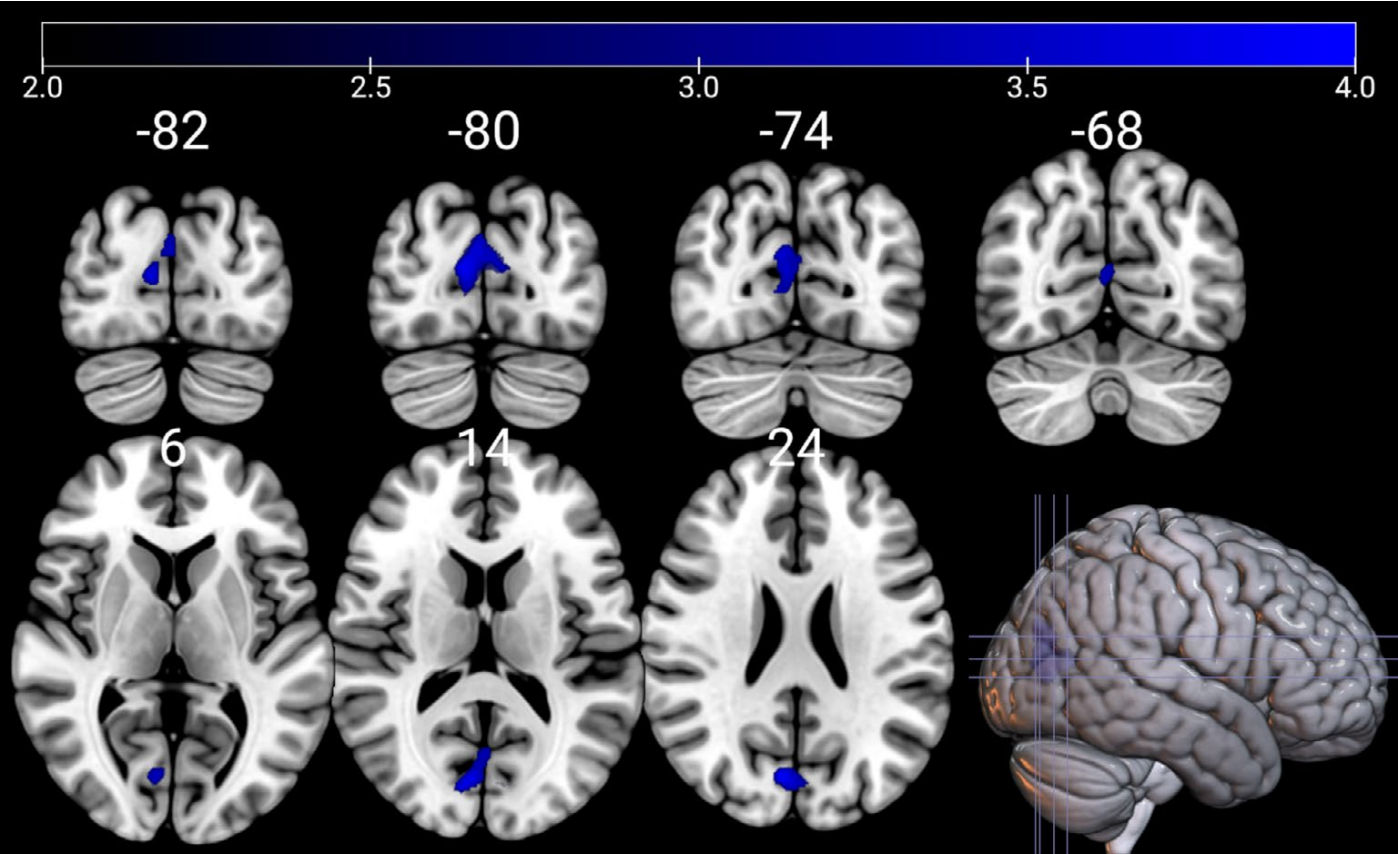
Positive association between maternal distress symptoms at 6 months postpartum and regional volume in the right medial occipital lobe, including the cuneus and calcarine regions, extending to the left hemisphere (controlling for key covariates and maternal distress symptoms at other ages). Only statistically significant regions at *p* < 0.001, false discovery rate corrected at cluster level (*p* < 0.05) are displayed. Color indicates *t*‐value. Images are in radiological orientation. Coordinates (indicated with white numbers) are in MNI space. The figure was created with MRIcroGL.

CDS at 24 months postpartum was negatively associated with GM volume in the left inferior/middle frontal gyrus (*p* = 0.011, cluster size 294, peak −40, 18, 18). The cluster did not remain significant after Bonferroni correction. The cluster is visualized in Figure 5. The cluster remains statistically significant in complete data analyses (*p* = 0.042, cluster size 123, peak −33, 18, 20) but not in winsorized analyses (*p* = 0.076, cluster size 131, peak −40, 18, 18). The same cluster was not observed in EPDS analyses but was observed in SCL analyses (*p* = 0.014, cluster size 198, peak −40, 16, 18).

**FIGURE 5:**
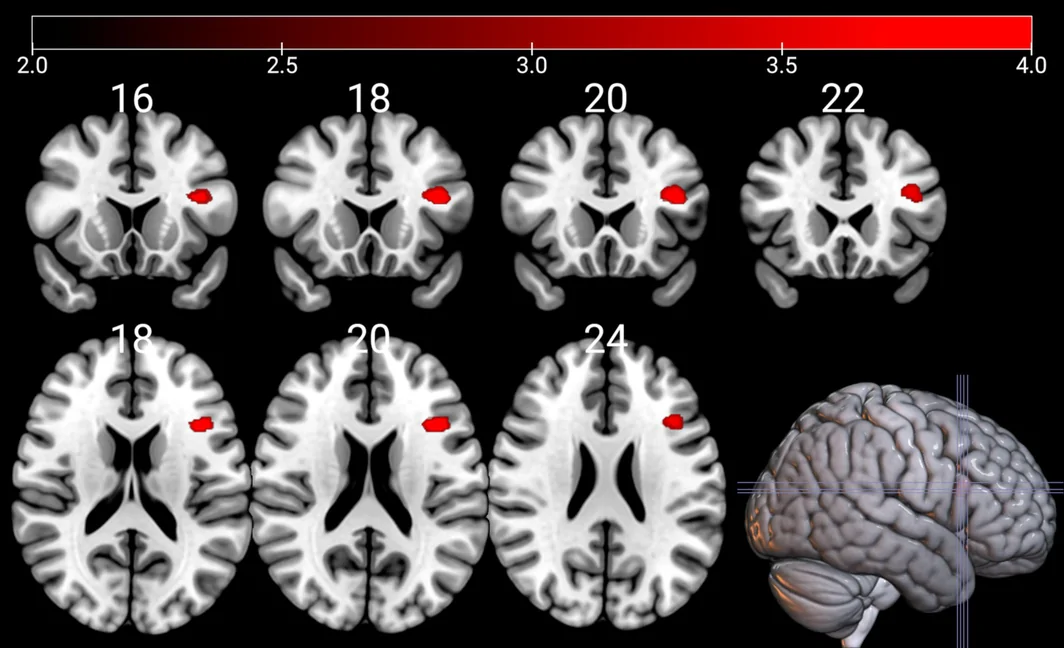
Negative association between maternal distress symptoms at 24 months postpartum and regional volume in the left inferior/middle frontal gyrus (controlling for key covariates and maternal distress symptoms at other ages). Only statistically significant regions at *p* < 0.001, false discovery rate corrected at cluster level (*p* < 0.05) are displayed. Color indicates *t*‐value. Images are in radiological orientation. Coordinates (indicated with white numbers) are in MNI space. The figure was created with MRIcroGL.

All significant clusters from the main analyses are summarized in Table 2.

**TABLE 2:** Clusters from the voxel‐based morphometry analysis.

Region	*x*	*y*	*z*	Cluster size	*p* (FDR‐corrected)	Peak *z*
**3 months postpartum key covariates (negative association)**						
Left supplementary motor cortex	−8	8	58	337	0.028	3.98
**3 months postpartum key covariates and distress at other ages (negative association)**						
Left supplementary motor cortex	−4	8	58	290	0.027	3.77
Right cerebellum (lobule VI)/occipital fusiform gyrus	32	−66	−20	467	0.010	3.82
Left anterior insula/frontal operculum	−39	10	2	353	0.018	3.67
**6 months postpartum key covariates and distress at other ages (positive association)**						
Right cuneus/calcarine cortex	9	−78	20	878	< 0.001	3.86
**24 months postpartum key covariates and distress at other ages (negative association)**						
Right supramarginal gyrus	−40	18	18	294	0.011	4.14
**6 months postpartum all covariates (positive association)**						
Right cuneus/calcarine cortex	9	−76	21	740	< 0.001	3.84
Right insula/operculum	45	−3	2	268	0.026	3.84
Right inferior occipital gyrus	45	−66	9	214	0.038	3.81
**24 months postpartum all covariates (negative association)**						
Left inferior/middle frontal gyrus	−40	18	18	193	0.044	3.90

*Note:* Region labels based on the neuromorphometrics atlas.

Abbreviation: FDR = false discovery rate.

#### 3.1.3 Controlling for All Covariates

Statistically significant results were observed at 6 and 24 months postpartum.

CDS at 6 months postpartum was positively associated with GM volume in the following regions: the right medial occipital lobe, including the cuneus and calcarine regions, extending to the left hemisphere (*p* < 0.001, cluster size 740, peak 9, −76, 21), the right insula/operculum (*p* = 0.026, cluster size 268, peak 45, −3, 2), and the right inferior occipital gyrus (*p* = 0.036, cluster size 214, peak 45, −66, 9). The right medial occipital lobe cluster remained significant after Bonferroni correction. Results are visualized in Figure 6. In complete data analyses, only the medial occipital lobe cluster remained significant (*p* = 0.004, cluster size 532, peak 8, −76, 24). Likewise, in winsorized analyses only the medial occipital lobe cluster remained significant (*p* = 0.002, cluster size 558, peak 8, −78, 20). EPDS analyses revealed the same medial occipital lobe cluster (*p* < 0.001, cluster size 1772, peak 9, −78, 16). SCL analyses revealed the same two clusters in the right insula/operculum (*p* = 0.017, cluster size 400, peak 45, −4, 2), and the right inferior occipital gyrus (*p* = 0.035, cluster size 285, peak 46, −64, 8).

**FIGURE 6:**
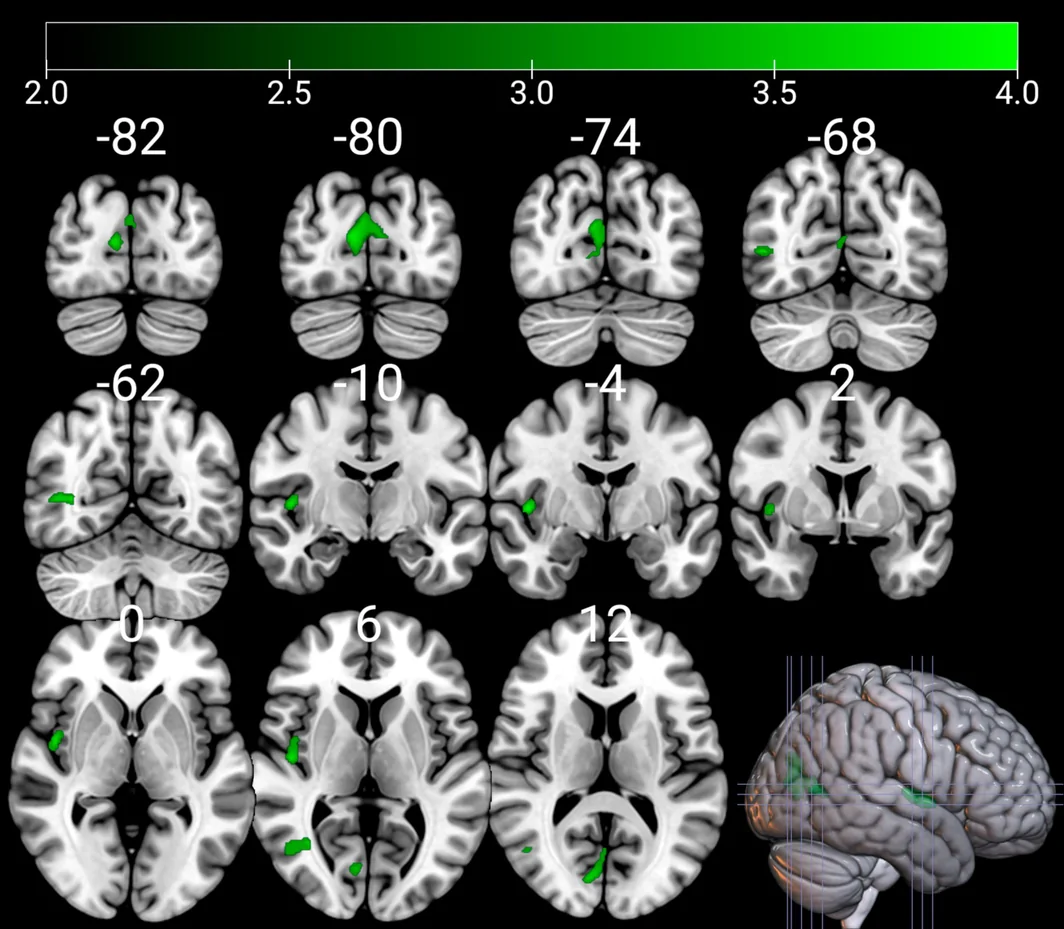
Positive associations between maternal distress symptoms at 6 months postpartum and regional volume in the right medial occipital lobe, including the cuneus and calcarine regions, extending to the left hemisphere, the right insula/operculum, and the right inferior occipital gyrus (controlling for all covariates). Only statistically significant regions at *p* < 0.001, false discovery rate corrected at cluster level (*p* < 0.05) are displayed. Color indicates *t*‐value. Images are in radiological orientation. Coordinates (indicated with white numbers) are in MNI space. The figure was created with MRIcroGL.

CDS at 24 months postpartum was negatively associated with GM volume in the left inferior/middle frontal gyrus (*p* = 0.044, cluster size 193, peak −40, 18, 18). The cluster did not remain significant after Bonferroni correction. The cluster is visualized in Figure 7. The cluster did not remain statistically significant in the complete data or winsorized analyses. Neither EPDS nor SCL analyses showed statistically significant clusters.

**FIGURE 7:**
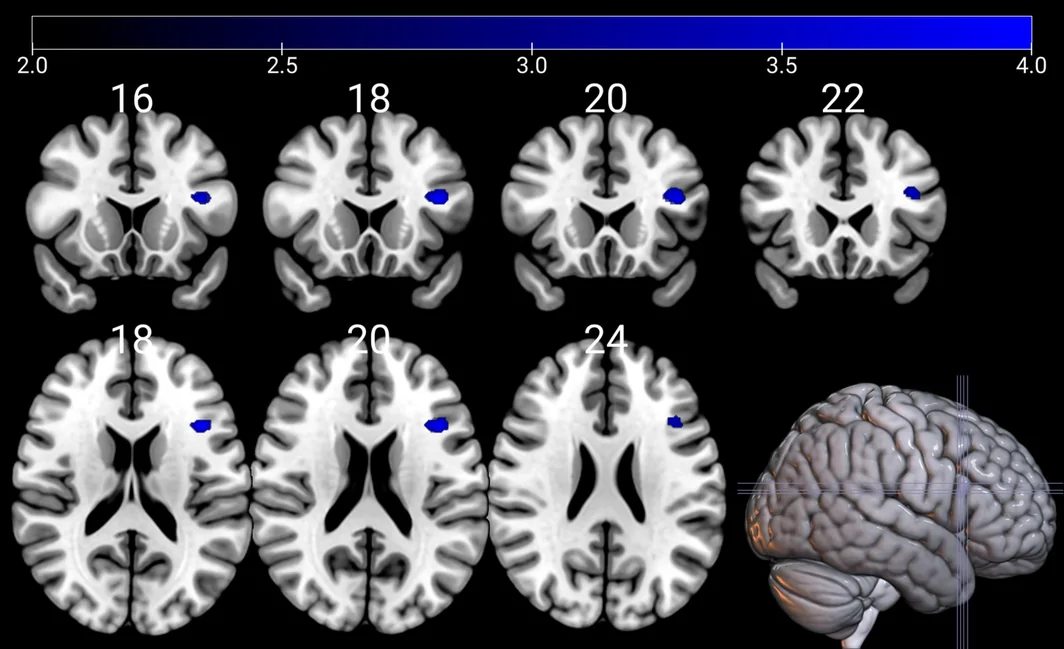
Negative association between maternal distress symptoms at 24 months postpartum and regional volume in the left inferior/middle frontal gyrus (controlling for all covariates). Only statistically significant regions at *p* < 0.001, false discovery rate corrected at cluster level (*p* < 0.05) are displayed. Color indicates *t*‐value. Images are in radiological orientation. Coordinates (indicated with white numbers) are in MNI space. The figure was created with MRIcroGL.

#### 3.1.4 Longitudinal Trajectories

A two‐class model was chosen (Table S3). It was the only model that had at least 5% of participants in each class, with 92.5% of participants in the low distress class and 7.5% in the increasing/high distress group. Model‐predicted trajectories and individual data are visualized in Figure S5. There were no statistically significant differences in brain structure between the groups.

#### 3.1.5 Post Hoc Analyses

The significant clusters from Table 2 were used as mediators between CDS from their corresponding time point and PIQ at 5 years of age. As expected, the association between brain cluster and corresponding CDS time point was significant (*p* < 0.05) in all 10 models. The total or direct effect of CDS on PIQ was never statistically significant (*p* > 0.05). A significant mediation effect was observed in one case: the right cerebellum/occipital fusiform gyrus cluster mediated the effect of distress exposure at 3 months postpartum on PIQ at 5 years (indirect effect = −0.1974, 95% CI −0.4443 to −0.0295). The total effect was nonsignificant (*B* = 0.0189, SE = 0.2871, *t* = 0.0659, *p* = 0.9475) and the direct effect was nonsignificant (*B* = 0.2164, SE = 0.2977, *t* = 0.7270, *p* = 0.4685), suggesting an indirect‐only mediation effect (Zhao et al. 2010). When the CI was changed to 99% to adjust for multiple comparisons, the indirect effect was no longer significant (99% CI −0.5400, 0.0184).

## 4 Discussion

This study investigated the association between pre‐ and postnatal maternal distress and child brain development at 5 years of age in the FinnBrain Birth Cohort study. We found that early postnatal maternal symptoms of depression and anxiety were associated with offspring brain morphology. The most prominent findings were found at 3 and 6 months of postnatal age. The associations with maternal distress were negative at 3 and 24 months, and positive at 6 months. Contrary to our hypotheses, no significant results were found in the hippocampus or amygdala. Finally, there was a surprising lack of prenatal findings, considering the previous extensive literature (Lautarescu, Craig, and Glover 2020).

Postnatal exposure to maternal distress was associated with widespread structural differences in the offspring's brain. The supplementary motor cortex (Nachev et al. 2008), middle frontal gyrus (Jung and Haier 2007), as well as the cerebellum (Buckner 2013; Vlasova et al. 2023; Zhang et al. 2023) have been associated with cognitive functioning. The cerebellar findings in this study were in lobule VI, which is involved in both motor and non‐motor functions (Guell and Schmahmann 2020). The medial occipital lobe volume and surface area were associated with nonverbal cognitive ability in our earlier study in the same population (Pulli et al. 2023). These findings indicate a possible connection between early‐life distress exposure and cognitive development. This connection has been studied following the 1998 ice storm in Québec, Canada (Laplante et al. 2004), wherein prenatal distress exposure explained over 10% of the variance in general cognitive ability and language skills in toddlerhood. Motor control is another aspect that connects to many of the regions in our study: the cerebellum, left supplementary motor cortex, and left anterior insula (Ackermann and Riecker 2004). Motor development has also been studied in the context of the Québec ice storm and there is some evidence of prenatal distress exposure affecting motor development in children (Cao et al. 2014). Our results indicate a potential connection between prenatal and early‐life distress exposure and cognitive and motor development. To make an initial investigation of this hypothesis, we performed post hoc analyses exploring the potential mediating role of brain structure between early distress exposure and cognitive ability at 5 years of age. There were no significant mediation, total, or direct effects. The role of brain development as a mediator between early exposures and individual developmental trajectories is worth exploring, and future studies are encouraged to study the mediating role of brain development within theory‐based exposure to outcome frameworks.

The direction of the associations changed between measurements, being negative at 3 and 24 months, and positive at 6 months postpartum. Especially between the temporally close 3‐ and 6‐month measurements, this difference seems surprising. It is important to note that the brain regions differ, and these regions reach their peak volume at different ages (Bethlehem et al. 2022). At 3 months, negative associations were observed in the supplementary motor cortex, fusiform gyrus, and anterior insula/frontal operculum, all of which are regions that reach their peak volume after 5 years, that is, lower volume reflects slower development under this interpretation. At 6 months, the main finding was the medial occipital lobe cluster, extending to multiple cortical regions, all of which reach their peak before 5 years of age, that is, larger volume reflects slower development. For the regions observed while controlling for all covariates, the inferior occipital region reaches peak volume at approximately the same time as the neuroimaging data was collected, while the insula/operculum region reaches its peak volume in later childhood. The findings mostly support the idea that early exposure to adversity (maternal depressive and anxiety symptoms) is associated with slower brain development, similar to that seen with another form of adversity, low SES, in prior research (Rakesh et al. 2023; Rakesh and Whittle 2021). In other words, results from different ages consistently contradict the stress acceleration hypothesis (Callaghan and Tottenham 2016). However, without repeated observations of the same participants, it is impossible to distinguish between slower and simply different development of specific brain regions following early distress exposure.

The hippocampus and amygdala have been associated with prenatal distress in multiple prior studies. Postnatal distress literature is more limited (Bezanson et al. 2023; Donnici et al. 2021; Wen et al. 2017). We found no significant clusters in either the hippocampus or the amygdala associated with either distress or depressive symptoms at any time point. The previous results have been inconsistent, and might be affected by the timing of exposure, or by the particular research questions (e.g., association only when looking at different genetic profiles (Qiu et al. 2017) or sexes (Lehtola et al. 2020) separately). Furthermore, the hippocampus and particularly the amygdala are small structures that are challenging to segment accurately (Lidauer et al. 2022), and methodological variation could also explain some of the inconsistent results.

We performed sensitivity analyses with depressive and anxiety symptoms, separately. The analyses with depressive symptoms replicated the supplementary motor cortex findings at 3 months of postnatal age and medial occipital lobe findings at 6 months of postnatal age, suggesting that those results were driven by depressive rather than anxiety symptoms. Anxiety symptoms, on the other hand, showed associations with local volumes in some regions that were not replicated in the composite distress analyses. Most importantly, the left middle temporal gyrus volume was negatively associated with anxiety symptoms at 3 months. Prenatal distress exposure has previously been associated with GM reductions in the bilateral middle temporal gyrus in children (Buss et al. 2010) and adolescents (Davis et al. 2020). The separate analyses with depressive symptoms and anxiety symptoms produced no overlapping results despite high intercorrelation.

Considering the high intercorrelation of the measurements, a supplementary trajectory‐based approach was implemented. There were no significant differences in brain structure between increasing/high and low distress groups. The lack of results is likely related to the vastly different sizes of the groups, which is related to the generally low levels of distress in our sample, as shown in our prior research with the larger FinnBrain Birth Cohort (Korja et al. 2024). Furthermore, the classes failed to capture prenatal and postnatal symptoms separately. One of the key findings of this study was the contrast between multiple postnatal findings and the absence of prenatal findings. The early postnatal period is a potential sensitive period for neural development, as it is characterized by neural processes like myelination and synaptogenesis (Silbereis et al. 2016) as well as a quick increase in cerebral GM volume (Bethlehem et al. 2022). Our results highlight the need to study the early postnatal period further, rather than just focus on pregnancy in studies focusing on early distress exposure.

This study has some limitations. There was some missing data, especially at the later time points. At the same time, including data from multiple time points is a key strength of the current study. There is a gap of a few years between exposure and brain measurement, and multiple family and environmental factors can shape neural development over this time period, not all of which can be addressed in this study. Furthermore, the data from the other parent is much more limited and were not included in this study, which means the results may partially reflect the broader home environment rather than the role of maternal distress, specifically. This study relied on self‐report questionnaires to assess maternal anxiety and depressive symptoms, which can be subject to measurement error. Despite the potential issues, self‐report remains the most common and widely accepted method for collecting data on subjective experiences, such as depressive and anxiety symptoms. This study used the standard preprocessing pipeline for VBM analyses, including registration to an adult MNI template, which requires more deformation of the child brain image and introduces an increased risk of errors (Fonov et al. 2011; Wilke et al. 2002). The images were visually quality controlled to rule out clear normalization errors and to mitigate the effects of the adult template. Finally, the homogeneity of our sample, consisting of FinnBrain participants mainly of Finnish origin from a specific region (i.e., Southwestern Finland), with a generally higher level of education, limits the generalizability of our findings to other populations and contexts. Replication in larger samples of varying ethnic backgrounds is recommended.

## 5 Conclusion

This study investigated the associations between maternal distress during the pre‐ and postnatal periods and the brain development of offspring at the age of 5 years. A particular strength of our work is the collection of distress measurements from multiple time points both pre‐ and postnatally. Our findings revealed associations between exposure to maternal distress at various developmental stages and structural alterations in widespread brain regions, particularly those associated with cognitive and motor functions. In contrast to prior literature, this study highlights the importance of the early postnatal period as a sensitive window for structural brain development. Importantly, structural associations with early‐life distress do not automatically indicate maladaptive development. These results form a basis for hypothesis creation for developmental pathways from early distress exposure to cognitive or behavioral outcomes. In conclusion, future studies should always consider the timing of the exposure, including the postnatal period, to the extent that they have available data.

## Funding

E.P.P. was supported by Strategic Research Council (SRC) established within the Research Council of Finland (#352648 and subproject #352655), Päivikki and Sakari Sohlberg Foundation, Juho Vainio Foundation, Emil Aaltonen Foundation, Finnish Brain Foundation, Turku University Foundation, Finnish Cultural Foundation, Signe and Ane Gyllenberg Foundation. S.A. was supported by the Signe and Ane Gyllenberg Foundation. A.R. was supported by the Signe and Ane Gyllenberg Foundation. H.K.A. was supported by the University of Turku Graduate School. P.J. was supported by the Finnish Brain Foundation, Signe and Ane Gyllenberg Foundation, Olvi Foundation, and Finnish Cultural Foundation. R.K. was supported by the Research Council of Finland (#308252). L.K. was supported by Finnish State Grants for Clinical Research (VTR), Signe and Ane Gyllenberg Foundation, Finnish Medical Foundation, and the Research Council of Finland (#308176, #308589).

## Ethics Statement

This study adheres to the ethical guidelines in the 1975 Declaration of Helsinki and has been approved by the joint Ethics Committee of the University of Turku and the Hospital District of Southwest Finland (ETMK:31/180/2011).

## Conflicts of Interest

The authors declare no conflicts of interest.

## Supporting information


**Figure S1:** Visualization of all the missing data in the final analysis sample (161 participants).
**Figure S2:** Negative associations between maternal distress symptoms at 3 months postpartum and regional volume in the left supplementary motor cortex, the right supramarginal gyrus, and the left anterior insula/operculum (controlling for key covariates, using winsorized data). Only statistically significant regions at *p* < 0.001, false discovery rate corrected at cluster level (*p* < 0.05) are displayed. Color indicates *t*‐value. Images are in radiological orientation. Coordinates (indicated with white numbers) are in MNI space. The figure was created with MRIcroGL.
**Figure S3:** Negative association between maternal anxiety symptoms at 3 months postpartum and regional volume in the left middle temporal gyrus (controlling for key covariates). Only statistically significant regions at *p* < 0.001, false discovery rate corrected at cluster level (*p* < 0.05) are displayed. Color indicates *t*‐value. Images are in radiological orientation. Coordinates (indicated with white numbers) are in MNI space. The figure was created with MRIcroGL.
**Figure S4:** Negative associations between maternal anxiety symptoms at 3 months postpartum and regional volume in the right cerebellum/occipital fusiform gyrus, the left middle temporal gyrus, and the right superior temporal/supramarginal gyrus (controlling for key covariates and other stress timepoints). Only statistically significant regions at *p* < 0.001, false discovery rate corrected at cluster level (*p* < 0.05) are displayed. Color indicates *t*‐value. Images are in radiological orientation. Coordinates (indicated with white numbers) are in MNI space. The figure was created with MRIcroGL.
**Figure S5:** Model‐predicted trajectories with participant data. Class 1 = low distress, Class 2 = increasing/high distress. CDS = Composite Distress Score. Time in weeks from first measurement (at gestational Week 14).
**Table S1:** STROBE checklist.
**Table S2:** Spearman's rho correlations between maternal depressive and anxiety symptoms at different age points.
**Table S3:** Model performance, latent class mixed model.

## Data Availability

Current EU and national legislation on personal data protection of sensitive data and the informed consents given by the study subjects do not permit open data sharing of research data collected in the FinnBrain Birth Cohort Study. Investigators interested in research collaboration and obtaining access to the data are encouraged to contact the FinnBrain board (finnbrain-board@lists.utu.fi). Contact information of the board members or FinnBrain's Principal Investigators is listed on the project website: https://sites.utu.fi/finnbrain/en/contact/.
