# Supplementary material for: Exposure to Maternal Pre‐ and Postnatal Psychological Distress: Associations With Brain Structure in 5‐Year‐Old Children
Source: Hum Brain Mapp. 2026 Nov 6;47(16):e70683. doi: 10.1002/hbm.70683 (PMC13743814; doi:10.1002/hbm.70683)
Supplement: Supplementary file 1 [file HBM-47-e70683-s001.docx]

# Exposure to Maternal Pre- and Postnatal Psychological Distress: Associations with Brain Structure in 5-year-old Children

Elmo P. Pulli, Hilyatushalihah K. Audah, Amira Svensk, Eeva Eskola, Elisabeth Nordenswan, Aylin Rosberg, Silja Luotonen, Pauliina Juntunen, Isabella L. C. Mariani Wigley, Venla Kumpulainen, Eero Silver, Anni Copeland, Ekaterina Saukko, Harri Merisaari, Riikka Korja, Eeva-Leena Kataja, Linnea Karlsson

# Methods and Materials

This study follows the Strengthening the Reporting of Observational Studies in Epidemiology (STROBE) guidelines (Vandenbroucke et al., 2014), see Table S1 for the attached checklist.

Table S1. STROBE checklist

|  | Item No | Recommendation | Location |
| --- | --- | --- | --- |
| **Title and abstract** | 1 | (*a*) Indicate the study’s design with a commonly used term in the title or the abstract | Abstract |
|  |  | (*b*) Provide in the abstract an informative and balanced summary of what was done and what was found | Abstract |
| Introduction | | | |
| Background/rationale | 2 | Explain the scientific background and rationale for the investigation being reported | Introduction |
| Objectives | 3 | State specific objectives, including any prespecified hypotheses | The last paragraph of “Introduction” |
| Methods | | | |
| Study design | 4 | Present key elements of study design early in the paper | Procedures |
| Setting | 5 | Describe the setting, locations, and relevant dates, including periods of recruitment, exposure, follow-up, and data collection | Participants and Supplementary materials |
| Participants | 6 | (*a*) Give the eligibility criteria, and the sources and methods of selection of participants | Participants and Supplementary materials |
| Variables | 7 | Clearly define all outcomes, exposures, predictors, potential confounders, and effect modifiers. Give diagnostic criteria, if applicable | Measures and Table 1 footnotes |
| Data sources/ measurement | 8 | For each variable of interest, give sources of data and details of methods of assessment (measurement). Describe comparability of assessment methods if there is more than one group | Measures and Image acquisition |
| Bias | 9 | Describe any efforts to address potential sources of bias | Supplementary materials |
| Study size | 10 | Explain how the study size was arrived at | Participants and Supplementary materials |
| Quantitative variables | 11 | Explain how quantitative variables were handled in the analyses. If applicable, describe which groupings were chosen and why | Data preprocessing and statistical analyses |
| Statistical methods | 12 | (*a*) Describe all statistical methods, including those used to control for confounding | Data preprocessing and statistical analyses |
|  |  | (*b*) Describe any methods used to examine subgroups and interactions | Statistics |
|  |  | (*c*) Explain how missing data were addressed | Measures |
|  |  | (*d*) If applicable, describe analytical methods taking account of sampling strategy | N/A |
|  |  | (*e*) Describe any sensitivity analyses | Statistics |
| Results | | | |
| Participants | 13 | (a) Report numbers of individuals at each stage of study—eg numbers potentially eligible, examined for eligibility, confirmed eligible, included in the study, completing follow-up, and analysed | Supplementary materials |
|  |  | (b) Give reasons for non-participation at each stage | N/A |
|  |  | (c) Consider use of a flow diagram | Participant |
| Descriptive data | 14 | (a) Give characteristics of study participants (eg demographic, clinical, social) and information on exposures and potential confounders | Table 1 |
|  |  | (b) Indicate number of participants with missing data for each variable of interest | Measures and Table 1 |
| Outcome data | 15 | Report numbers of outcome events or summary measures | Table 1 and Supplementary materials |
| Main results | 16 | (*a*) Give unadjusted estimates and, if applicable, confounder-adjusted estimates and their precision (eg, 95% confidence interval). Make clear which confounders were adjusted for and why they were included | No unadjusted estimates. Main adjusted results under “Results” |
|  |  | (*b*) Report category boundaries when continuous variables were categorized | N/A |
|  |  | (*c*) If relevant, consider translating estimates of relative risk into absolute risk for a meaningful time period | N/A |
| Other analyses | 17 | Report other analyses done—eg analyses of subgroups and interactions, and sensitivity analyses | Results and Supplementary materials |
| Discussion | | | |
| Key results | 18 | Summarise key results with reference to study objectives | First paragraph of Discussion |
| Limitations | 19 | Discuss limitations of the study, taking into account sources of potential bias or imprecision. Discuss both direction and magnitude of any potential bias | Last paragraph of Discussion |
| Interpretation | 20 | Give a cautious overall interpretation of results considering objectives, limitations, multiplicity of analyses, results from similar studies, and other relevant evidence | Discussion |
| Generalisability | 21 | Discuss the generalisability (external validity) of the study results | Last paragraph of Discussion |
| Other information | | | |
| Funding | 22 | Give the source of funding and the role of the funders for the present study and, if applicable, for the original study on which the present article is based | Financial Support |

## Participants

The participants are a part of the prospective FinnBrain Birth Cohort Study ([www.finnbrain.fi](http://www.finnbrain.fi)), which examines the influence of genetic and environmental factors on child development and later health outcomes (Karlsson et al., 2018). Pregnant women (n = 3808) attending their first trimester ultrasound at gestational week (GW) 12, their spouses (n = 2623), and children (n = 3837; including 29 twin pairs) were recruited in Southwest Finland between December 2011 and April 2015. An ultrasound-verified pregnancy and sufficient knowledge of Finnish or Swedish languages were required for participation. The cohort study includes several follow-up studies.

For the neuroimaging visit, we primarily recruited participants that had attended the neuropsychological visit. The participants recruited for both visits were focus cohort families (Karlsson et al., 2018) and families who had actively participated in previous FinnBrain study visits. For the neuroimaging visits, 541 families were contacted and 478 (88.4%) of them were reached. In total, 203 (37.5%) participants attended imaging visits (113 boys (55.7%), mean age 5.40 (SD 0.13), range 5.08–5.79 years). For this study, we included only participants with an adequate quality T1 image, assessed by the author E.P.P. in our prior work (Pulli et al., 2022), resulting in the final sample size of 173.

We originally aimed to scan all subjects between the ages 5 years 3 months and 5 years 5 months; however, there was a pause in visits due to the start of the COVID-19 pandemic, and subsequently many of the participants were older than planned when they were scanned (152/203 (74.9%) of the participants attended the visit within the intended age range). The exclusion criteria for the neuroimaging study were: 1) born before GW 35 (before GW 32 for those with exposure to maternal prenatal synthetic glucocorticoid treatment), 2) developmental anomaly or abnormalities in senses or communication (e.g., blindness, deafness, congenital heart disease), 3) known long-term medical diagnosis (e.g., epilepsy, autism), 4) ongoing medical examinations or clinical follow up in a hospital (meaning there has been a referral from primary care setting to special health care), 5) child use of continuous, daily medication (including per oral medications, topical creams and inhalants; One exception to this was desmopressin medication, which was allowed), 6) history of head trauma (defined as concussion necessitating clinical follow up in a health care setting or worse), 7) metallic (golden) ear tubes (to assure good-quality scans), and routine magnetic resonance imaging (MRI) contraindications.

## Bias assessment

Mothers of the children who did not participate in the neuropsychological visits (out of the 1288 contacted families) had a lower education level (χ2(2) = 30.94, p < 0.001), a lower monthly income (χ2(3) = 11.65, p = 0.009) and were younger (t (1286) = -4.130, p < 0.001) compared to the mothers in the families that participated in the neuropsychological visits.

Mothers of the children who participated in the neuropsychological visits but not in the neuroimaging visits were older (t (369) = 1.97, p = 0.047) but did not differ in education level or monthly income compared to the mothers in the families that participated in the MRI visit.

## Measures

**Depressive symptoms**: To assess maternal depressive symptoms, the Finnish version of the Edinburgh Postnatal Depression Scale (EPDS)(Cox et al., 1987) was used. EPDS is a 10-item self-report screening tool that is widely used to identify depressive symptoms in women both during and after pregnancy. The participant is asked to report their symptoms from the past seven days. Each item is rated on a 4-point Likert scale (0 = never or not at all, 3 = very often or most of the time), with total scores ranging from 0 to 30. The clinical cut-off threshold for potential depression is ≥ 10 points.

**Anxiety symptoms**: Maternal anxiety symptoms were evaluated using the Finnish version of the Symptom Checklist-90-Revised (SCL)(Derogatis, 1994). SCL is a self-report questionnaire comprising of 90 questions, designed to assess a wide range of psychiatric and somatic symptoms. However, for the purposes of this study, only the anxiety subscale was utilized. The 10-item scale assesses signs of nervousness, tension, and feelings of panic. Items are scored on a 5-point scale assessing the level of distress a given symptom has caused in the past month (0 = not at all, 4 = extremely), with total scores ranging from 0 to 40 and higher scores indicating more symptoms.

The EPDS and SCL scores were not standardized while calculating the CDS, as that would make the effect sizes not comparable with other studies (Pearson correlations between non-standardized and standardized CDS = 1.000 at each timepoint; Spearman correlation > 0.998 at each timepoint).

A correlation matrix of EPDS and SCL scores from all age points is presented in Table S2. Missing data is visualized in Figure S1. Visualized with R version 4.3.1 in RStudio (Version 2022.07.1+554)(RStudio Team, 2020).

Table S2. Spearman’s rho correlations between maternal depressive and anxiety symptoms at different age points

|  | **EPDS 14 GW** | **EPDS 24 GW** | **EPDS 34 GW** | **EPDS 3 mo** | **EPDS 6 mo** | **EPDS 24 mo** | **SCL 14 GW** | **SCL 24 GW** | **SCLs 34 GW** | **SCL 3 mo** | **SCL 6 mo** |
| --- | --- | --- | --- | --- | --- | --- | --- | --- | --- | --- | --- |
| **EPDS 14 GW** | -- |  |  |  |  |  |  |  |  |  |  |
| **EPDS 24 GW** | .566^***^ | -- |  |  |  |  |  |  |  |  |  |
| **EPDS 34 GW** | .552^***^ | .634^***^ | -- |  |  |  |  |  |  |  |  |
| **EPDS 3 mo** | .498^***^ | .521^***^ | .418^***^ | -- |  |  |  |  |  |  |  |
| **EPDS 6 mo** | .453^***^ | .514^***^ | .394^***^ | .671^***^ | -- |  |  |  |  |  |  |
| **EPDS 24 mo** | .269^**^ | .344^***^ | .364^***^ | .534^***^ | .522^***^ | -- |  |  |  |  |  |
| **SCL 14 GW** | .688^***^ | .441^***^ | .378^***^ | .389^***^ | .289^***^ | .195^*^ | -- |  |  |  |  |
| **SCL 24 GW** | .577^***^ | .665^***^ | .504^***^ | .516^***^ | .442^***^ | .284^**^ | .668^***^ | -- |  |  |  |
| **SCL 34 GW** | .538^***^ | .492^***^ | .597^***^ | .394^***^ | .364^***^ | .266^**^ | .657^***^ | .734^***^ | -- |  |  |
| **SCL 3 mo** | .443^***^ | .455^***^ | .385^***^ | .590^***^ | .406^***^ | .316^***^ | .541^***^ | .553^***^ | .562^***^ | -- |  |
| **SCL 6 mo** | .466^***^ | .534^***^ | .469^***^ | .594^***^ | .706^***^ | .419^***^ | .472^***^ | .551^***^ | .578^***^ | .661^***^ | -- |
| **SCL 24 mo** | .253^**^ | .303^***^ | .307^***^ | .432^***^ | .449^***^ | .593^***^ | .376^***^ | .343^***^ | .357^***^ | .445^***^ | .472^***^ |

*** = p < 0.001, ** = p < 0.01, * = p < 0.05. EPDS = Edinburgh Postnatal Depression Scale, SCL = Symptom Checklist, gwks = gestational weeks, mo = months. Spearman correlations calculated without data imputation. Number of missing data points: 14 GW n = 7, 24 GW n = 6, 34 GW n = 6; 3 months n = 16, 6 months n = 30, 24 months n = 42.

Figure S1. Visualization of all the missing data in the final analysis sample (161 participants).

## Cognitive performance measurement

The neuropsychological study visits for 5-year-old children included neurocognitive testing, eye-movement tracking, mother-child interaction assessment, and questionnaires filled out by the parents. Neurocognitive testing included assessments of the child’s general cognitive ability (WPPSI-III subtests Block Design, Matrix Reasoning and Similarities) and executive functioning and self-regulation, of which only the non-verbal tasks from the general cognitive ability assessments are used in the current study.

The approximately two-hour-long study visits were conducted and video recorded by graduate students in quiet examination rooms and the data collection was overseen by PhD students/psychologists. The graduate students were trained by PhD students/psychologists prior to data collection, to ensure unified test administration among all students, and to ensure that the students had sufficient interaction skills to scaffold the children’s motivation and mood during the study visit. Written informed consent was provided by the parents prior to the study visit, and the parents received feedback of the child’s performance on some of the assessment methods after the study visit.

Non-verbal ability was assessed using the Finnish version of WPPSI-III, which is a standardized and widely used measure of cognitive ability in young children from ages 2 years and 6 months to 7 years and 3 months (Wechsler, 2009). In this study, a composite sum score of non-verbal ability (PIQ; mean 100) was estimated using two subtests: the Block Design task measuring visuospatial ability and the Matrix Reasoning task measuring visual abstract reasoning. The standardized scale scores corresponding the raw scores of the subtests were based on Finnish norms and result in a mean of 10, reflecting standardized mean performance in the population at each age. Additionally, analyses of the subtests were conducted separately to get further information on the possible subtest driving the findings.

## Neuroimaging visits

MRI scanning was conducted at Turku University Hospital by research staff. Prior to the visits, families were contacted and the purpose of the study, along with the scanning procedure, were explained to the parents. Parents were then requested to share this information with their child and inquire whether they would like to participate. Follow-up phone calls were made by a research nurse to confirm the willingness to participate. Written informed consent was obtained from both parents prior to any experimental procedures. In preparation for the MRI imaging visits, a member of the research team met with each participating family in person. The families were provided with practice materials to help the children become familiar with the imaging procedure and to facilitate practice sessions for scanning at home. On the day of the MRI, a simulated scan was conducted in the laboratory using a homemade non-commercial wooden coil to further familiarize the child with the scanning process. Additionally, as is customary in MRI studies involving children, a light meal was provided before the actual experimental scans took place (A. M. Copeland et al., 2021).

Participants underwent scanning either during natural sleep or awake while watching a movie/TV show of their preference. Earplugs and headphones were provided for hearing protection, and foam padding was applied to reduce head movement and enhance comfort during scanning. Throughout the scanning process, a member of the research team and the child’s parent(s) stayed in the scanner room. Participants were given a signal ball that they could use to pause or stop the scan at any point if needed. More detailed information regarding the protocol and study visits is presented in previous publications (A. Copeland et al., 2022; Pulli et al., 2022).

# Results

## Voxel-based morphometry results – sensitivity analyses

The significant clusters that were present in sensitivity analyses, but not main analyses are presented here. There were findings from all three main models.

### Controlling for key covariates

Statistically significant results were observed at 3 months postpartum in the winsorized and SCL analyses. Winsorized analyses showed three clusters with negative associations with CDS at 3 months postpartum: the left supplementary motor cortex (p = 0.001, cluster size 744, peak -8, 8, 58), the right supramarginal gyrus (p = 0.013, cluster size 351, peak 58, -28, 48), and the left anterior insula/operculum (p = 0.008, cluster size 432, peak -42, 8, 10). Clusters are visualized in Figure S2. SCL analyses revealed a negative association between SCL at 3 months postpartum and GM volume in the left middle temporal gyrus (p = 0.037, cluster size 330, peak -68, -10, -16). The cluster is visualized in Figure S3.

Figure S2. Negative associations between maternal distress symptoms at 3 months postpartum and regional volume in the left supplementary motor cortex, the right supramarginal gyrus, and the left anterior insula/operculum (controlling for key covariates, using winsorized data). Only statistically significant regions at p < 0.001, false discovery rate corrected at cluster level (p < 0.05) are displayed. Color indicates t-value. Images are in radiological orientation. Coordinates (indicated with white numbers) are in MNI space. The figure was created with MRIcroGL.

Figure S3. Negative association between maternal anxiety symptoms at 3 months postpartum and regional volume in the left middle temporal gyrus (controlling for key covariates). Only statistically significant regions at p < 0.001, false discovery rate corrected at cluster level (p < 0.05) are displayed. Color indicates t-value. Images are in radiological orientation. Coordinates (indicated with white numbers) are in MNI space. The figure was created with MRIcroGL.

### Controlling for other distress timepoints

SCL analyses revealed a negative association between SCL at 3 months postpartum and GM volume in three clusters: the right cerebellum / occipital fusiform gyrus (p = 0.004, cluster size 474, peak 26, -72, -14), the left middle temporal gyrus (p = 0.001, cluster size 706, peak -68, -9, -16), and the right superior temporal / supramarginal gyrus (p = 0.004, cluster size 468, peak 70, -32, 20). The clusters are visualized in Figure S4.

Figure S4. Negative associations between maternal anxiety symptoms at 3 months postpartum and regional volume in the right cerebellum / occipital fusiform gyrus, the left middle temporal gyrus, and the right superior temporal / supramarginal gyrus (controlling for key covariates and other stress timepoints). Only statistically significant regions at p < 0.001, false discovery rate corrected at cluster level (p < 0.05) are displayed. Color indicates t-value. Images are in radiological orientation. Coordinates (indicated with white numbers) are in MNI space. The figure was created with MRIcroGL.

### Longitudinal trajectories

Model-predicted trajectories and individual data are visualized in Figure S5. The big (92.5 %) Class 1 showed low distress, while the small (7.5 %) Class 2 showed higher levels of distress overall, but also a trend of increasing distress from pre- to postnatal period.

Table S3. Model performance, latent class mixed model.

| Groups | Log-likelihood | AIC | BIC | SABIC | Entropy | Class % |
| --- | --- | --- | --- | --- | --- | --- |
| 1 | -2714.27 | 5440.54 | 5459.03 | 5440.03 | 1 | 100 |
| 2 | -2688.02 | 5394.05 | 5421.78 | 5393.29 | 0.921 | 93/7 |
| 3 | -2669.19 | 5362.38 | 5399.36 | 5361.37 | 0.923 | 14/83/4 |
| 4 | -2655.58 | 5341.17 | 5387.39 | 5339.91 | 0.915 | 4/79/3/14 |
| 5 | -2646.25 | 5328.50 | 5383.96 | 5326.98 | 0.902 | 2/71/6/4/17 |

Abbreviations: AIC = Akaike Information Criterion, BIC = Bayesian Information Criterion, SABIC = Sample-size Adjusted BIC.

Figure S5. Model-predicted trajectories with participant data. Class 1 = low distress, Class 2 = increasing/high distress. CDS = Composite Distress Score. Time in weeks from first measurement (at gestational week 14).

# References

Copeland, A., Korja, R., Nolvi, S., Rajasilta, O., Pulli, E. P., Kumpulainen, V., Silver, E., Saukko, E., Hakanen, H., Holmberg, E., Kataja, E.-L., Häkkinen, S., Parkkola, R., Lähdesmäki, T., Karlsson, L., Karlsson, H., & Tuulari, J. J. (2022). Maternal sensitivity at the age of 8 months associates with local connectivity of the medial prefrontal cortex in children at 5 years of age. *Frontiers in Neuroscience*, *16*, 1560. https://doi.org/10.3389/FNINS.2022.920995

Copeland, A. M., Silver, E. A. O., Korja, R., Lehtola, S., Merisaari, H., Saukko, E., Sinisalo, S., Saunavaara, J., Lähdesmäki, T., Parkkola, R., Nolvi, S., Karlsson, L., Karlsson, H., & Tuulari, J. (2021). Infant and child MRI: a review of scanning procedures. *Frontiers in Neuroscience*, *15*, 632. https://doi.org/10.3389/FNINS.2021.666020

Cox, J. L., Holden, J. M., & Sagovsky, R. (1987). Detection of Postnatal Depression. *British Journal of Psychiatry*, *150*(06), 782–786. https://doi.org/10.1192/bjp.150.6.782

Derogatis, L. R. (1994). SCL-90-R: Administration, scoring and procedures manual (3rd ed.). *NCS Pearson.*

Karlsson, L., Tolvanen, M., Scheinin, N. M., Uusitupa, H.-M., Korja, R., Ekholm, E., Tuulari, J. J., Pajulo, M., Huotilainen, M., Paunio, T., & Karlsson, H. (2018). Cohort Profile: The FinnBrain Birth Cohort Study (FinnBrain). *International Journal of Epidemiology*, *47*(1), 15–16j. https://doi.org/10.1093/ije/dyx173

Pulli, E. P., Silver, E., Kumpulainen, V., Copeland, A., Merisaari, H., Saunavaara, J., Parkkola, R., Lähdesmäki, T., Saukko, E., Nolvi, S., Kataja, E.-L., Korja, R., Karlsson, L., Karlsson, H., & Tuulari, J. J. (2022). Feasibility of FreeSurfer Processing for T1-Weighted Brain Images of 5-Year-Olds: Semiautomated Protocol of FinnBrain Neuroimaging Lab. *Frontiers in Neuroscience*, *16*, 640. https://doi.org/10.3389/FNINS.2022.874062

RStudio Team. (2020). RStudio: Integrated Development for R. *RStudio, PBC, Boston*. http://www.rstudio.com/

Vandenbroucke, J. P., von Elm, E., Altman, D. G., Gøtzsche, P. C., Mulrow, C. D., Pocock, S. J., Poole, C., Schlesselman, J. J., Egger, M., Blettner, M., Boffetta, P., Brenner, H., Chêne, G., Cooper, C., Davey-Smith, G., Gagnon, F., Greenland, P., Greenland, S., Infante-Rivard, C., … Zou, G. Y. (2014). Strengthening the Reporting of Observational Studies in Epidemiology (STROBE): Explanation and elaboration. *International Journal of Surgery*, *12*(12), 1500–1524. https://doi.org/10.1016/J.IJSU.2014.07.014

Wechsler, D. (2009). WPPSI-III - Wechsler Preschool And Primary Scale Of Intelligence - Third Edition. *Psykologien Kustannus Oy, Helsinki*.
